# Development of a risk prediction model for infection-related mortality in patients undergoing peritoneal dialysis

**DOI:** 10.1371/journal.pone.0213922

**Published:** 2019-03-20

**Authors:** Hiroaki Tsujikawa, Shigeru Tanaka, Yuta Matsukuma, Hidetoshi Kanai, Kumiko Torisu, Toshiaki Nakano, Kazuhiko Tsuruya, Takanari Kitazono

**Affiliations:** 1 Department of Medicine and Clinical Science, Kyushu University, Fukuoka, Japan; 2 Fukuoka Dental College, Fukuoka, Japan; 3 Kokura Memorial Hospital, Fukuoka, Japan; 4 Department of Integrated Therapy for Chronic Kidney Disease, Kyushu University, Fukuoka, Japan; 5 Department of Nephrology, Nara Medical University, Nara, Japan; University of Wisconsin, UNITED STATES

## Abstract

**Background:**

Assessment of infection-related mortality remains inadequate in patients undergoing peritoneal dialysis. This study was performed to develop a risk model for predicting the 2-year infection-related mortality risk in patients undergoing peritoneal dialysis.

**Methods:**

The study cohort comprised 606 patients who started and continued peritoneal dialysis for 90 at least days and was drawn from the Fukuoka Peritoneal Dialysis Database Registry Study in Japan. The patients were registered from 1 January 2006 to 31 December 2016 and followed up until 31 December 2017. To generate a prediction rule, the score for each variable was weighted by the regression coefficients calculated using a Cox proportional hazard model adjusted by risk factors for infection-related mortality, including patient characteristics, comorbidities, and laboratory data.

**Results:**

During the follow-up period (median, 2.2 years), 138 patients died; 58 of them of infectious disease. The final model for infection-related mortality comprises six factors: age, sex, serum albumin, serum creatinine, total cholesterol, and weekly renal Kt/V. The incidence of infection-related mortality increased linearly with increasing total risk score (P for trend <0.001). Furthermore, the prediction model showed adequate discrimination (c-statistic = 0.79 [0.72–0.86]) and calibration (Hosmer–Lemeshow test, P = 0.47).

**Conclusion:**

In this study, we developed a new model using clinical measures for predicting infection-related mortality in patients undergoing peritoneal dialysis.

## Introduction

Infectious diseases are often life-threatening and are one of the leading causes of death in patients undergoing peritoneal dialysis (PD) [1–3]. The survival of patients undergoing PD or hemodialysis has improved during the last 20 years [4, 5]. However, the risk of infection-related mortality remains higher in patients undergoing PD than in the general population [6]. Therefore, it is important for clinicians to identify patients undergoing PD who have a high risk of infection-related mortality to improve their survival rate.

Many previous studies have identified risk factors for infection-related mortality in patients undergoing PD, including age, diabetes mellitus, serum potassium, serum albumin, serum creatinine, serum phosphate, and other variables [1, 7–12]. Identification of patients at high risk of infection-related mortality may facilitate the development of targeted intervention strategies for improving outcomes. Nevertheless, previous studies have not yet addressed the extent to which various clinical variables affect the risk of infection-related death in patients undergoing PD.

Prediction models for predicting the risk of all-cause and cardiovascular mortality among patients undergoing hemodialysis and PD have recently been developed [13–19]. However, there has been limited research regarding infection-related death in patients undergoing PD. This study aimed to develop and verify the internal validity of a new clinical risk prediction model for predicting infection-related mortality within 2 years after registration among patients undergoing PD.

## Materials and methods

### Design

The participants in this retrospective multicenter cohort study were also included in part of our previous multicenter cohort study (Fukuoka Peritoneal Dialysis Database Study). Eriguchi et al. reported that use of an extended swan-neck catheter with an upper abdominal exit site reduces the incidence of PD-related infections, and Tsuruya et al. reported a positive association between residual kidney function (RKF) and hemoglobin concentration in this cohort [20, 21]. The present study protocol was approved by the Local Ethics Committee of Kyushu University Hospital (No. 21–16), registered with the University Hospital Medical Information Network (UMIN000018902), and performed according to the ethics of clinical research described in the Declaration of Helsinki. Written informed consent was obtained from each patient prior to their participation in the study. If informed consent was not acquired, opt-out consent to participation was obtained through the study website.

### Participants

The study cohort comprised 606 patients who had undergone PD for at least 90 days at seven dialysis facilities in Fukuoka prefectures in Kyushu, Japan. The patients were registered from 1 January 2006 to 31 December 2016 and followed until they switched to hemodialysis, received a kidney transplant, died during PD, were lost to follow-up, or until 31 December 2017.

### Outcomes

The end point of the study was infection-related death after registration. Data were collected from the patients’ medical records. Patients were censored if they had been switched to hemodialysis or renal transplantation, died of a non-infection-related condition, were lost to follow-up, or were still alive at the end of follow-up. All mortality events were retrieved from the medical records and carefully examined. When patients moved to other dialysis facilities where there were no collaborators of this study, information regarding their health condition by mail.

### Clinical variables

The patient characteristics assessed in this study were age and sex. The clinical data were body mass index, cause of end-stage renal disease (diabetic nephropathy or other conditions), history of cardiovascular disease, duration of PD, systolic blood pressure, cardiothoracic ratio, hemoglobin, serum albumin, peritoneal and weekly renal Kt/V urea, blood urea nitrogen, creatinine (Cr), serum potassium, serum calcium, serum phosphorus, and total cholesterol.

### Statistical analysis

Continuous parametric data are expressed as mean ± standard deviation, continuous nonparametric data as median and interquartile range, and categorical data as frequency. The univariate hazard ratio (HR) with 95% confidence interval (95% CI) was estimated for each risk factor of infection-related mortality using a Cox proportional hazards model. To generate the risk prediction model, independent risk factors for infection-related mortality were selected using a multivariate Cox proportional hazards model analysis with backward stepwise selection and P <0.05 for all variables. The final model comprised six variables: age, sex, serum albumin, serum Cr, total cholesterol, and weekly renal Kt/V. The score for each variable was weighted according to the estimated regression coefficient of the final Cox model. This method is based on the method of Sullivan, et al. [22].

To develop a simple integer-based point score for each variable, each β coefficient was divided by the model's minimum coefficient value (excluding β factors of <0.05) and rounded up to the nearest integer to assign a score [23]. The 2-year absolute risk of incident infection-related death predicted by the total risk score was computed using a Cox proportional hazards model with the baseline survival function. Internal validity and discriminative ability were assessed using c-statistics and calibration was examined by the Hosmer–Lemeshow test. For all tests, a P-value of <0.05 was considered to denote statistical significance. All statistical analyses were performed using SAS software package version 9.4 (SAS Institute, Cary, NC, USA) and R version 3.0.2 (R Foundation for Statistical Computing).

## Results

### Characteristics and clinical features of study participants

The patients’ characteristics are shown in Table 1. Their median age was 65 years, and 68.5% of them were men. Diabetic nephropathy was present in 55.6% of all patients. The median weekly renal Kt/V at study entry was 0.63.

**Table 1:** Patients’ baseline characteristics (N = 606).

Age (years)	65 (56–74)
Men (%)	68.5
PD duration (months)	6 (3–10)
Diabetic nephropathy (%)	55.6
Past history of CVD (%)	19.8
Height (cm)	161.3 (153.9–167)
Body weight (kg)	61.3 (52.7–67.8)
body mass index (kg/m^2^)	23.2 (21.3–25.7)
Weekly peritoneal Kt/V	1.02 (0.82–1.25)
Weekly renal Kt/V	0.63 (0.35–0.94)
Systolic blood pressure (mmHg)	135 (120–151)
Diastolic blood pressure (mmHg)	77 (66–88)
PD volume (mL)	4500 (4500–6000)
Use of icodextrin (%)	59.1
Serum total protein (g/dL)	6.2 (5.7–6.6)
Serum albumin (g/dL)	3.3 (2.9–3.5)
Serum Cr (mg/dL)	7.9 (5.8–9.7)
Blood urea nitrogen (mg/dL)	53.7 (45.2–64.1)
Uric acid (mg/dL)	6.1 (5.2–6.9)
Serum calcium (mg/dL)	8.4 (7.8–8.9)
Serum phosphorus (mg/dL)	4.6 (4–5.5)
Aspartate transaminase (U/L)	18 (13–24)
Alanine transaminase (U/L)	13 (9–20)
Serum sodium (mEq/L)	137 (135–140)
Serum potassium (mEq/L)	4.2 (3.7–4.7)
Serum chloride (mEq/L)	98 (95–101)
Total cholesterol (mg/dL)	180 (155–207)
Hemoglobin (g/dL)	10.3 (9.4–11.2)
Cardiothoracic ratio (%)	51.3 (46.8–55.7)

Data are expressed as mean ± standard deviation (continuous parametric data), median and interquartile range (continuous nonparametric data), or frequency (categorical data).

PD, peritoneal dialysis; CVD, cardiovascular disease; Cr, creatinine.

### Development of model for predicting risk of infection-related mortality

The median duration of follow-up after registration in this study was 2.2 years. During follow-up, 138 patients (22.8%) died. Infection was the most common cause of death in the cohort, occurring in 58 patients (42.0%), the most common cause of infection-related death being pneumonia (43.1%). Cardiovascular-related death, the second most common cause of death, was documented in 48 patients (34.8%), followed by tumor-related death in 13 patients (9.4%) and malnutrition-related death in nine (6.5%) (Tables 2 and 3).

**Table 2:** All-cause mortality.

	N (patients)	%
CVD-specific death	48	34.8
Infection-related death	58	42.0
Tumor-related death	13	9.4
Malnutrition-related death	9	6.5
Others	10	7.2
Total deaths	138	100

CVD, cardiovascular disease.

**Table 3:** Infection-related mortality.

	N (patients)	%
Pneumonia	25	43.1
Peritonitis	6	10.3
Sepsis	8	13.8
Cellulitis	7	12.1
Others	12	20.7
Total infection-related death	58	100

Eleven variables (age, sex, history of cardiovascular disease, cardiothoracic ratio, dialysate volume, serum albumin, blood urea nitrogen, Cr, serum potassium, serum phosphorus, and total cholesterol) were significantly associated with a higher risk of infection-related mortality according to univariate analysis (Table 4). The following six clinical variables were selected as independent risk factors for infection-related death by multivariate analysis with backward stepwise elimination: age (HR, 1.06; 95% CI, 1.03–1.09), weekly renal Kt/V (HR, 2.66; 95% CI, 1.29–5.47), serum albumin (HR, 1.89; 95% CI, 1.08–3.31), total cholesterol (HR, 1.01; 95% CI, 1.00–1.02), serum Cr (HR, 1.17; 95% CI, 1.02–1.33), and male sex (HR, 1.93; 95% CI, 1.00–3.73) (Table 5).

**Table 4:** Unadjusted HRs for infection-related mortality.

	HR	P value
Age (1-year increase)	1.08 (1.05–1.11)	<0.001
Men (vs. Women)	1.95 (1.05–3.62)	0.034
Diabetic nephropathy	0.84 (0.49–1.44)	0.534
Past history of CVD	2.46 (1.44–4.21)	<0.001
PD duration (1-month increase)	0.99 (0.98–1.01)	0.486
Body mass index (1-kg/m^2^ decrease)	1.02 (0.94–1.12)	0.589
Systolic blood pressure (10-mmHg increase)	0.99 (0.98–1.00)	0.180
Cardiothoracic ratio (1% increase)	1.04 (1.00–1.08)	0.038
Dialysate volume (100-mL decrease)	1.03 (1.01–1.05)	0.008
Use of icodextrin	0.86 (0.51–1.43)	0.555
Weekly renal Kt/V (1-unit decrease)	0.55 (0.29–1.06)	0.074
PD Kt/V (1-unit increase)	0.77 (0.36–1.66)	0.508
Serum albumin (1-g/dL decrease)	0.28 (0.17–0.46)	<0.001
Blood urea nitrogen (10-mg/dL decrease)	0.75 (0.62–0.91)	0.003
Serum Cr (1-mg/dL decrease)	0.82 (0.74–0.91)	<0.001
Serum potassium (1-mEq/L decrease)	0.57 (0.39–0.85)	0.005
Serum calcium (1-mg/dL decrease)	0.79 (0.59–1.05)	0.109
Serum phosphorus (1-mg/dL increase)	0.73 (0.56–0.95)	0.020
Total cholesterol (10-mg/dL increase)	0.89 (0.83–0.95)	<0.001
Hemoglobin (1-g/dL increase)	1.11 (0.92–1.33)	0.270
Past peritonitis	1.12 (0.55–2.28)	0.753

HR, hazard ratio; CVD, cardiovascular disease; PD, peritoneal dialysis; Cr, creatinine.

**Table 5:** Multivariate-adjusted HRs for infection-related mortality.

	HR	P value
Age (1-year increase)	1.06 (1.03–1.09)	<0.001
Serum albumin (1-g/dL decrease)	1.89 (1.08–3.31)	<0.026
Serum Cr(1-mg/dL decrease)	1.17 (1.02–1.33)	<0.019
Total cholesterol (10-mg/dL decrease)	1.11 (1.02–1.20)	0.014
Weekly renal Kt/V (0.1-unit decrease)	1.10 (1.03–1.19)	<0.001
Male (vs. Female)	1.93 (1.00–3.73)	0.049

HR, hazard ratio; Cr, creatinine. Variables (age, serum albumin, serum Cr, total cholesterol, weekly renal Kt/V, and sex) were selected using a Cox proportional hazard model and a stepwise backward method with P < 0.05 for the remaining variables to determine the risk factors for infection-related death.

### Creating a Score-Based Prediction Rule

**Fig 1:**
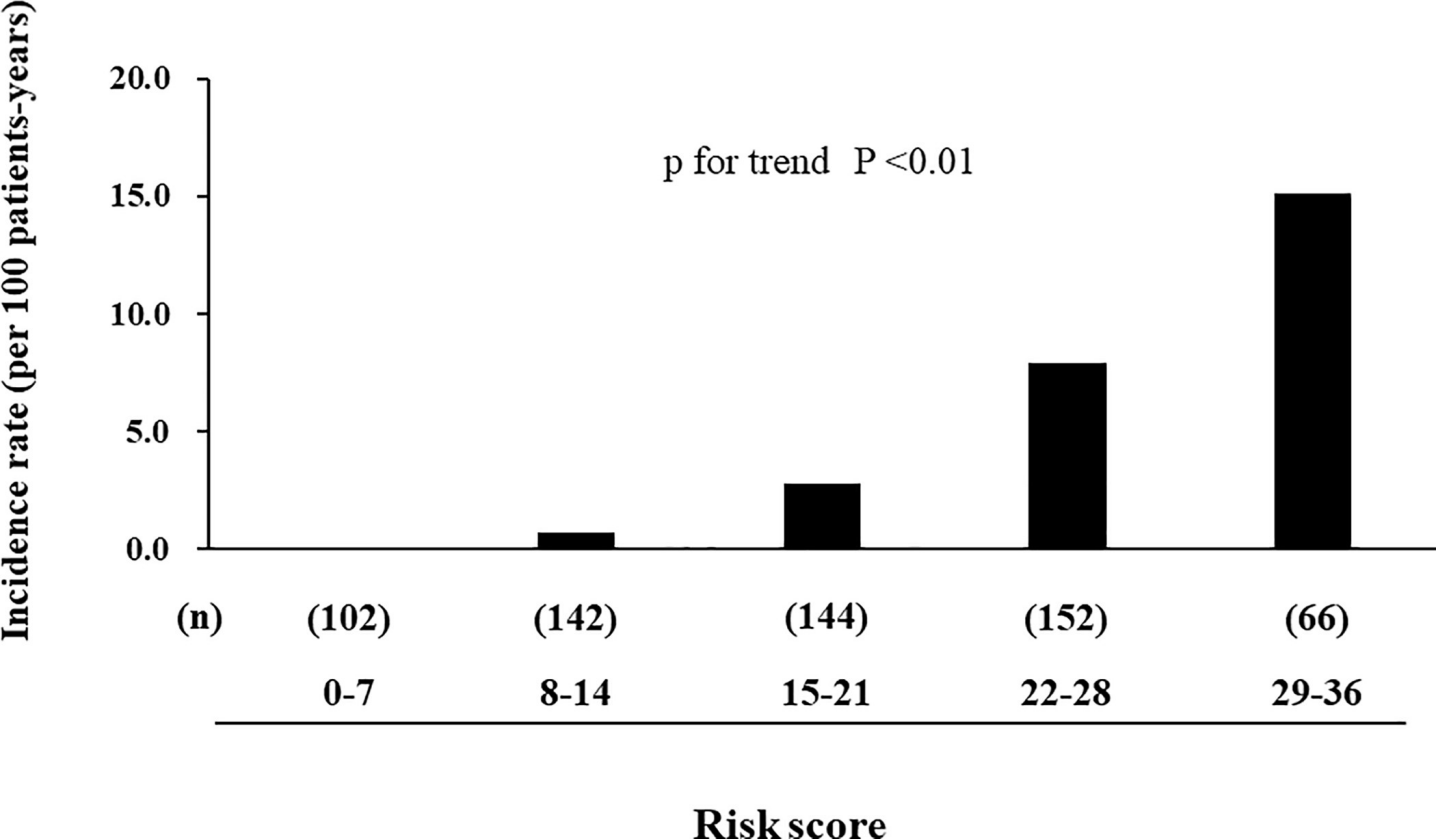
Incidence of infection-related death by increments of total risk score.

**Fig 2:**
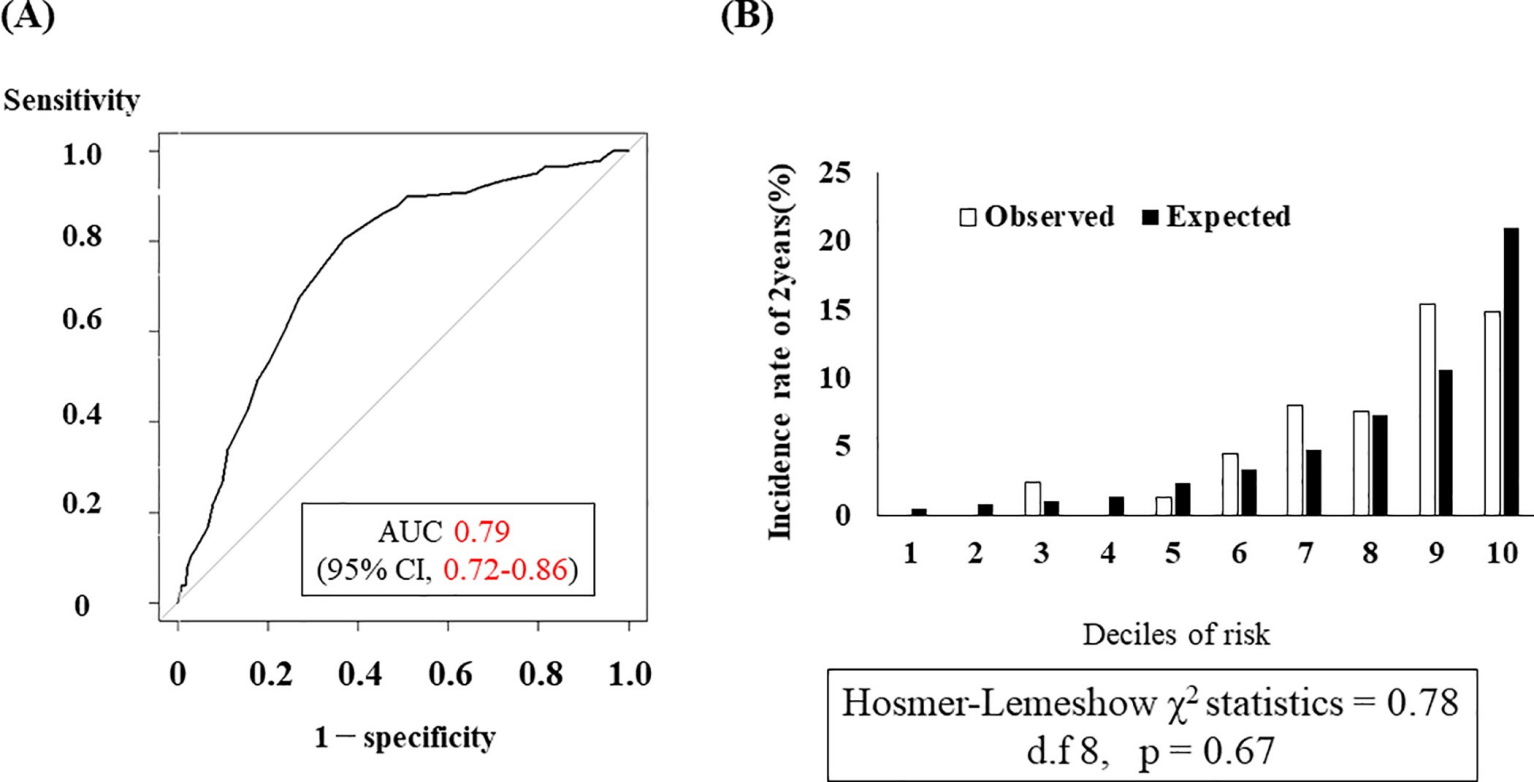
Evaluation of internal validity of risk model. (A) C-statistics among the risk prediction models using only the final score. (B) Observed and predicted 2-year absolute risk for development of infection-related death by deciles of risk. Hosmer–Lemeshow χ^2^ statistic = 0.78, d.f. = 8, P = 0.67.

A score-based prediction rule comprising six variables was created (Table 6). One point in the prediction rule corresponded to 0.175, which was the minimum regression coefficient (<0.05 values were ignored) in the selected variables (Table 7). The risk of infection-related death increased 1.15-fold (95% CI, 1.10 to 1.21) for each 1-point in the total risk score. The predicted 2-year absolute risks of infection-related mortality per 1-point increase in the total prediction rule are shown in Table 8. The incidence of infection-related death increased linearly as the total risk score increased (P for trend <0.01) (Fig 1). Our prediction rule performed moderately well in terms of discrimination for predicting 2-year infection-related mortality with a c-statistic of 0.79 (95% CI, 0.72–0.86) and showed adequate calibration as assessed by the Hosmer–Lemeshow test (χ^2^ statistic with 0.78, d.f. = 8, P = 0.67) (Fig 2). Additionally, our subgroup analysis showed there was no difference in c-statistics between the group with BMI 27 or more and the group with lower BMI (S1 Fig). Furthermore, the same variables were applied to the prediction models for overall mortality or CVD-specific mortality. The prediction model for overall mortality showed adequate discrimination (c-statistic = 0.76 [0.72–0.80]) and calibration (Hosmer–Lemeshow test, P = 0.12). However, the model for CVD-specific mortality did not show adequate discrimination (c-statistic = 0.68 [0.60–0.76]). (S2 Fig). Finally, a prediction model using the same statistical analysis for CVD-specific death was developed. The selected variables were age, cardiothoracic ratio, past history of CVD, PD duration, and systolic blood pressure. The prediction model using these variables for CVD-specific mortality did not show adequate discrimination (c-statistic = 0.71 [0.65–0.78]) (S3 Fig).

**Table 6:** Multivariate-adjusted HRs for infection-related mortality using categorical variables.

			Multivariate-adjusted model		
	N (patients)		HR	P value	*β*
Age (year)	226	≤60	1		Ref
	170	61–70	3.08 (1.15–8.25)	0.026	1.124
	210	≥71	6.90 (2.65–17.95)	<0.001	1.932
Serum albumin (g/dL)	447	≥3.0	1		Ref
	159	<3.0	2.7 (1.58–4.63)	<0.001	0.995
Serum Cr (mg/dL)	299	≥8	1		Ref
	307	<8	1.24 (0.66–2.34)	0.506	0.216
Total cholesterol (mg/dL)	309	≥180	1		Ref
	297	<180	1.71 (0.97–3.01)	0.063	0.537
Weekly renal Kt/V	174	≥0.80	1		Ref
	219	0.40–0.79	1.15 (0.61–2.15)	0.666	0.138
	213	<0.40	1.65 (0.81–3.34)	0.169	0.498
Sex	191	F	1		Ref
	415	M	1.86 (0.97–3.56)	0.062	0.619

HR, hazard ratio; Cr, creatinine.

**Table 7:** Risk scores for infection-related mortality.

Age (year)		Score
	≤60	0 points
	61–70	8 points
	≥71	15 points
Serum albumin (g/dL)		
	≥3.0	0 points
	<3.0	7 points
Serum Cr (mg/dL)		
	≥8	0 points
	<8	2 points
Total cholesterol (mg/dL)		
	≥180	0 points
	<180	4 points
Weekly renal Kt/V		
	≥0.80	0 points
	≥0.40	1 points
	<0.40	4 points
Sex		
	F	0 points
	M	4 points

Cr, creatinine.

**Table 8:** The predicted 2-year absolute risks of infection-related mortality.

Score	Predicted 2-Year Absolute Risk(%)	Score	Predicted 2-Year Absolute Risk(%)
0	0.42	19	5.63
1	0.48	20	6.43
2	0.55	21	7.35
3	0.63	22	8.39
4	0.73	23	9.57
5	0.83	24	10.91
6	0.96	25	12.43
7	1.10	26	14.13
8	1.26	27	16.05
9	1.44	28	18.20
10	1.66	29	20.60
11	1.90	30	23.26
12	2.18	31	26.22
13	2.50	32	29.47
14	2.86	33	33.03
15	3.28	34	36.89
16	3.75	35	41.05
17	4.30	36	45.49
18	4.92		

## Discussion

We here developed a new prediction rule for calculating the 2-year absolute risk of infection-related mortality in patients undergoing PD. This prediction rule has appropriate discriminative ability to identify patients likely to develop future infection-related death. Additionally, the estimated incidence calculated by our prediction model demonstrated good fit with the observed incidence in this cohort. In particular, all variables in our prediction rule are related to protein-energy wasting (PEW), which emphasizes a strong association between PEW and the risk of infectious disease-related death.

This prediction rule consists of six variables: age, sex, serum Cr, serum albumin, total cholesterol, and weekly renal Kt/V. The prognostic factors age, serum Cr, serum albumin, total cholesterol, and RKF are consistent with the findings of many prior investigators [9, 10, 12, 24–29]. Additionally, prior investigators have reported that diabetes mellitus is an important prognostic factor for infectious complications [8]. However, in the present study, adding "presence/absence of diabetic nephropathy" to the covariates of the developed model did not improve discrimination. It is possible that the severity of the diabetes mellitus, as HbA1c, is an important factor. Further studies are needed to elucidate this association. Our prediction rule includes comprehensively prognostic factors for patients undergoing PD and the variables were selected by a statistically rigorous method. Additionally, the absolute risk of future infection-related death is estimated by combining these plausible risk factors, and the degree of influence on the prognosis of each factor is weighted by the score. These are important features of our model.

Several risk scores for predicting all-cause mortality and cardiovascular mortality in patients undergoing dialysis have been reported previously [13–19]. One study has already developed and validated a risk model for cardiovascular mortality in patients undergoing PD. In that study, age, BMI, blood pressure, serum lipids, fasting glucose, sodium, albumin, total protein, and phosphorus were the strongest predictors [17]. However, these studies did not focus on cause-specific mortality, such as that related to infection. Because infection is one of the leading causes of death in patients undergoing PD, we focused on infection-related death. To the best of our knowledge, our study represents the first attempt to develop a risk score for calculating absolute risk of infection-related mortality in patients undergoing PD. The rates of hospitalization because of infection are increasing [3], and mortality secondary to sepsis is approximately 50-fold higher in patients undergoing dialysis than in the general population [2]. Therefore, early identification of patients at high risk of infection-related mortality is crucial to delaying or preventing death. We consider that this score will be valuable for selecting therapeutic strategies for patients undergoing PD.

All variables incorporated in the prediction rule (age, sex, serum Cr, serum albumin, total cholesterol, and weekly renal Kt/V) are associated with PEW [12, 26, 30–35], emphasizing a strong association between PEW and the risk of infectious disease-related death. It has been well established that malnutrition is associated with all-cause, cardiovascular, and infection-related mortality in patients undergoing PD [10, 36]. However, in our study, there were weak associations between those variables and CVD- specific death (S2 Fig). Furthermore, we developed our prediction model using the same statistical analysis for CVD-specific death. However, the prediction model for CVD-specific mortality did not show adequate discrimination (S3 Fig). Because there were fewer CVD-specific than infection-related deaths, we had insufficient statistical power to perform a reliable analysis. Further studies are needed to develop a prediction model for CVD-specific mortality in patients undergoing PD. PEW, which is caused by a hypercatabolic status, uremic toxins, malnutrition, and inflammation, is exceptionally common and closely associated with mortality and morbidity in patients with end-stage renal disease [37]. Hypoalbuminemia and hypocholesterolemia are criteria for the clinical diagnosis of PEW [34]. Previous studies have shown that serum Cr is derived from skeletal muscle and may serve as a biomarker of somatic body protein in patients undergoing PD [35] and that higher serum Cr levels are associated with better survival [12]. RKF is independently associated with greater intake of dietary protein, calories, and other nutrients [31], and patients with preserved RKF have a better nutritional status [32, 33]. These effects of RKF may help to reduce inflammation and uremic toxins [38]. Older adults are more susceptible to malnutrition than younger individuals [29]. In addition, men may be more susceptible to uremia than women to inflammation-induced anorexia [39], and inflammatory and nutritional variables may deteriorate over time in men [40]. Whether male sex is risk factor for infection-related mortality is controversial [41–44]. However, our findings may partly explain the sex-related differences in the risk of infection-related mortality in patients undergoing PD. Compared with patients undergoing hemodialysis in the USA [45], all-cause mortality was low (69 vs. 208.3 per 1000 patients), whereas infection-related death occurred more frequently. Additionally, the proportion of diabetic nephropathy was higher, the participants were older, and BMI was lower in our study. Thus, we have confirmed that this model is valid in patients with high BMIs (S1 Fig). Our subgroup analysis suggested that this prediction model is useful regardless of BMI. As described above, our rule comprises reliable clinical variables that are routinely examined.

This study has several limitations. First, we did not verify the external validity of our risk score in another independent validation cohort. Therefore, the application of our prediction rule to other patient groups may be limited. Second, the misclassification that can occur with one-time measurement of each risk factor potentially weakens the associations found in this study. Third, we were unable to obtain information about smoking habits. Patients with a smoking habit undergoing PD are reportedly at greater risk of mortality [46, 47]. Thus, information about smoking is of great importance, especially regarding infection-related mortality. Another topic for future research is examination of the relationship between PEW and infection-related mortality taking the influence of smoking into account. Fourth, the participating patients in this study were Japanese; thus this model may require adaptation for other patient cohorts. Finally, this was a retrospective study and therefore, has inherent limitations and possible selection bias. Despite these limitations, we believe that this study is valuable in that it is the first to develop a risk score for infection-related mortality in patients undergoing PD.

In conclusion, we have developed a new prediction rule with for calculating the absolute risk of infection-related mortality over a 2-year period in patients undergoing PD. This rule comprises readily available and clinically reliable factors associated with PEW and may serve as an important guide in identifying patients undergoing PD who have a higher risk of infection-related mortality. Further research is needed to determine whether therapeutic interventions that are developed according to this prediction rule will improve the prognosis of patients undergoing PD.

## Supporting information

S1 Fig Subgroup analysis stratified by BMI. (A) C-statistics stratified by BMI<27 among the risk prediction models for all-cause mortality using final model
(B) C-statistics stratified by BMI≥27 among the risk prediction models for all-cause mortality using final model.
(TIF)

S2 Fig Internal validity of risk prediction model for all-cause and CVD specific mortality using the same variables. (A) C-statistics among the risk prediction models for all-cause mortality using the same variables
(B) C-statistics among the risk prediction models for CVD-specific mortality using the same variables.
(TIF)

S3 Fig Internal validity of risk prediction model for CVD-specific mortality using the same statistical analysis. C-statistics among the risk prediction models for CVD-specific mortality using the same statistical analysis.
(TIF)
