# Supplementary figures and images for: Development of a risk prediction model for infection-related mortality in patients undergoing peritoneal dialysis
Source: PLoS One. 2019 Mar 20;14(3):e0213922. doi: 10.1371/journal.pone.0213922 (PMC6426225; doi:10.1371/journal.pone.0213922)

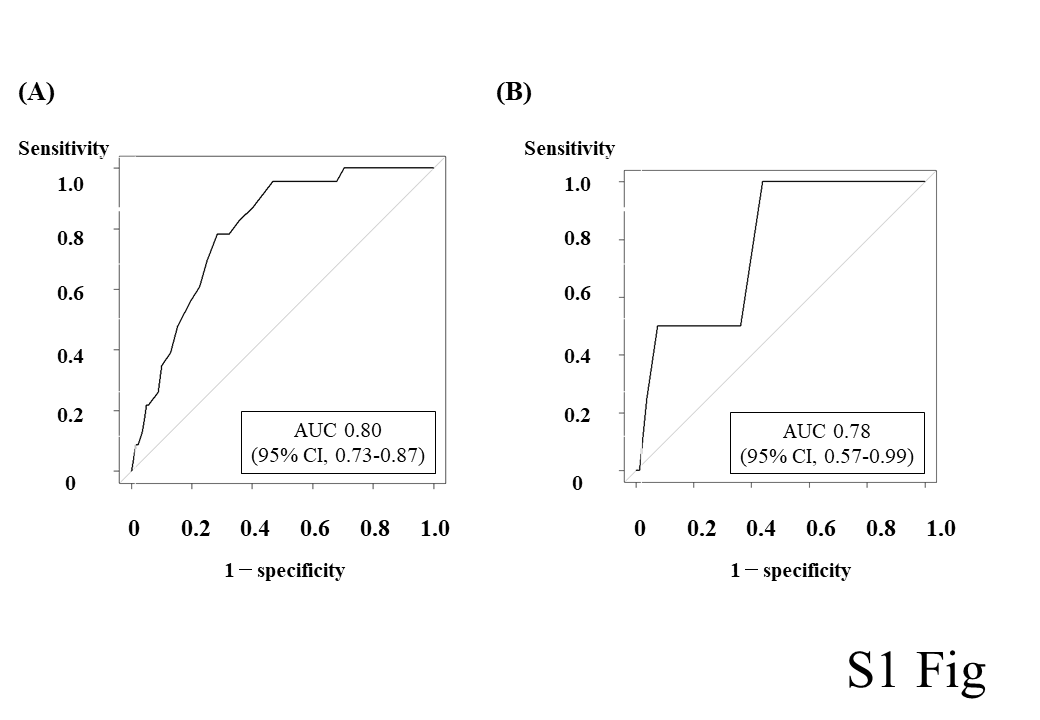

Supplement: S1 Fig — (A) C-statistics stratified by BMI<27 among the risk prediction models for all-cause mortality using final model (B) C-statistics stratified by BMI≥27 among the risk prediction models for all-cause mortality using final model. (TIF) [file pone.0213922.s001.TIF]

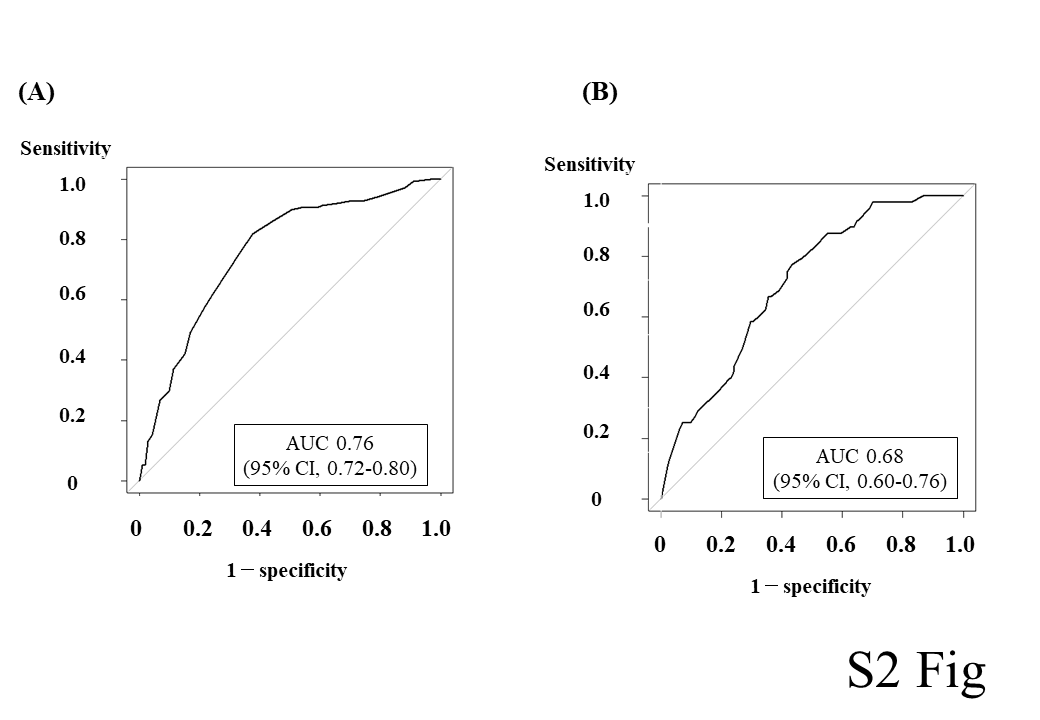

Supplement: S2 Fig — (A) C-statistics among the risk prediction models for all-cause mortality using the same variables (B) C-statistics among the risk prediction models for CVD-specific mortality using the same variables. (TIF) [file pone.0213922.s002.TIF]

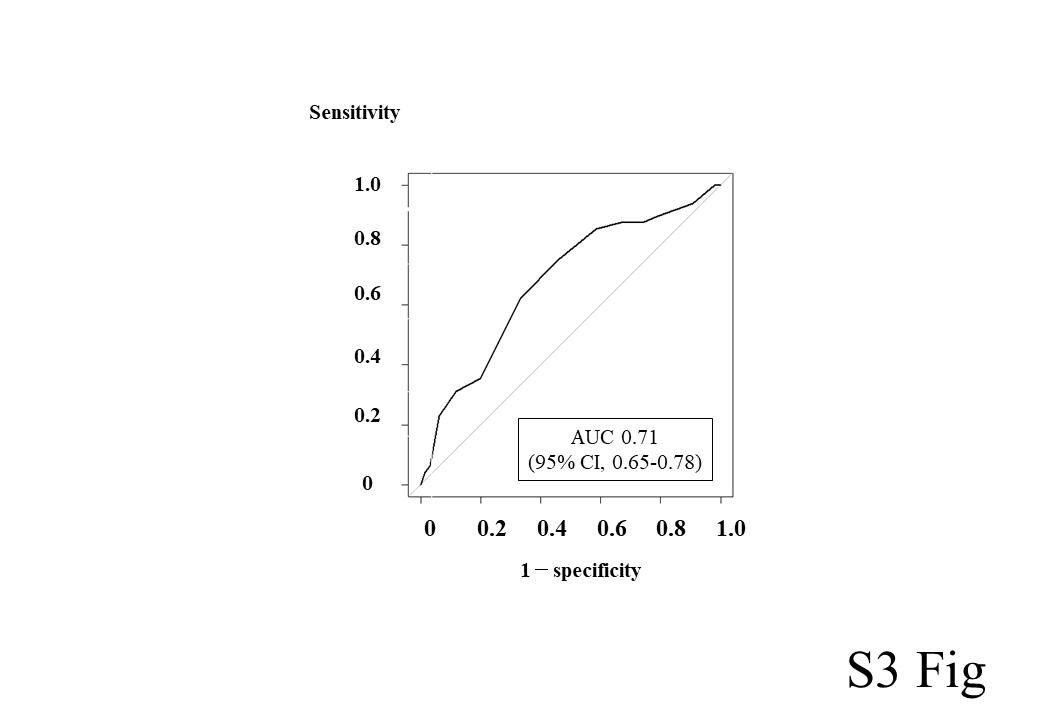

Supplement: S3 Fig — C-statistics among the risk prediction models for CVD-specific mortality using the same statistical analysis. (TIF) [file pone.0213922.s003.TIF]
